# 119. A Humanized Antibody Targeting the CotH Invasins is Protective Against Murine Mucormycosis

**DOI:** 10.1093/ofid/ofab466.119

**Published:** 2021-12-04

**Authors:** Yiyou Gu, Abdullah Alqarihi, Shakti Singh, Teclegiorgis Gebremariam, Sondus Alkhazraji, Eman Youssef, David Andes, Ashraf S Ibrahim

**Affiliations:** 1 The Lundquist Institute, Torrance, California; 2 The Lundquist Institute at Harbor-UCLA Medical Center, Torrance, California; 3 Department of Medicine at University of Wisconsin, Madison, WI, Torrance, California; 4 University of Wisconsin, Madison, Wisconsin; 5 David Geffen School of Medicine, Torrance, California

## Abstract

**Background:**

Despite antifungal therapy and surgical debridement, overall mortality of invasive mucormycosis is >40%. Currently the world is witnessing an explosion in mucormycosis in India among COVID-19 patients with an official count of 28,252 cases as of 06/07/2021. Thus, novel therapeutic modalities are needed. We previously reported on a mouse monoclonal antibody (C2) targeting CotH invasins being protective against mucormycosis. Here, we humanized C2 MAb and assessed its efficacy *in vitro* and *in vivo.*

**Methods:**

The C2 (IgG1) paratopes of the heavy chain and light chain were grafted on the most suitable human IgG1 with back mutations in the paratopes needed to restore binding of humanized clones to CotH3 (by biolayer interferometry using Gator). Clones were compared to C2 in their ability to prevent *Rhizopus delemar*-induced injury to A549 alveolar epithelial and primary human endothelial cells and for enhancing human neutrophil killing of the fungus *in vitro*. C2 and the humanized clones were also compared for their ability to protect neutropenic mice from mucormycosis induced by *R. delemar* or *Mucor cicrinelloides* with and without antifungal therapy.

**Results:**

Three humanized clones showed 10-fold enhanced binding affinity to CotH3 protein (~5 nM for humanized *vs.* ~50 nM for C2). One humanized clone (VX01) doubled the ability of neutrophils to kill *R. delemar* and resulted in ~50% reduction in host cell damage. A single low dose of VX01 (30 µg) given 24 h post infection resulted in comparable survival of 60-70% in mice infected intratracheally with either *R. delemar* or *M. cicrinelloides vs.* placebo mice (0% survival, *P* < 0.02). Importantly, VX01 acted synergistically in protecting mice when combined with liposomal amphotericin B or posaconazole in a severe model of mucormycosis with treatment starting 48 h post infection (~70% survival for combination *vs.* 0-20% survival for monotherapy and reduced lung fungal burden by 1.5 log, *P*< 0.001). GLP-tissue cross reactivity studies of VX01 showed favorable safety profiles.

**Conclusion:**

VX01 shows enhanced binding to CotH3 protein and maintained the protective features of C2 MAb against murine mucormycosis. Clinical testing of combination therapy of VX01 + antifungals is warranted. VX01 is currently in manufacturing.

**Disclosures:**

**Yiyou Gu, PhD**, **Vitalex Biosciences** (Shareholder) **Ashraf S. Ibrahim, PhD**, **Vitalex Biosciences** (Shareholder)

